# To: Predictors of coronary artery disease in cardiac arrest survivors: coronary angiography for everyone? A single-center retrospective analysis

**DOI:** 10.5935/0103-507X.20220030-en

**Published:** 2022

**Authors:** Ignacio Barriuso, Patricia Irigaray, Kristian Rivera, Diego Fernández-Rodríguez

**Affiliations:** 1 Hospital Universitari Arnau de Vilanova - Lleida, España.

**TO THE EDITOR**

We congratulate Rigueira et al.^(1)^ for their interesting study on the predictors of significant coronary artery disease (CAD) in sudden cardiac arrest (SCA) survivors to establish the best timing for coronary angiography and determine the relationship between CAD, percutaneous coronary intervention (PCI) and mortality.

One hundred seventeen patients were included, highlighting that 68.4% of patients presented significant CAD; defined as stenosis of ≥ 50% for the left main artery and 70% for the remaining vessels. ST-segment elevation and wall motion abnormalities were identified as independent predictors of significant CAD. However, neither the presence of significant CAD nor PCI performance was associated with mortality, and we were unable to establish the optimal timing for coronary angiography.^(1)^>

The TOMAHAWK trial^(2)^ has renewed interest in coronary angiography in SCA. In this regard, the present study^(1)^ provides a valuable discussion, integrating study information with scientific evidence in a complex field of research such as SCA. Nevertheless, we point out some of the following considerations:

1. Angiographically significant CAD: Our principal objection to the investigation stems from diagnosing significant CAD exclusively using angiographic assessments.^(1)^ Extensive evidence demonstrates that the functional repercussions of intermediate coronary stenosis (ranging from 30 to 90%) are highly variable, showing that a 70% cutoff for lumen reduction could be misclassified as inducing-ischemia stenoses in acute coronary syndromes.^(3)^ In the absence of intravascular coronary imaging permitting an accurate identification of culprit lesions, providing information about angiographic features suggestive of unstable plaques, such as ulceration, filling defects consistent with thrombus or impaired flow, could be helpful to link CAD with SCA.^(4)^

2. Defining the cause of SCA: Traditionally, ischemic heart disease has been blamed for the vast majority of SCA cases. However, recent works have described a reduction in SCA attributable to ischemic heart disease.^(5)^ The etiologic diagnosis after SCA is challenging due to patients’ inability to communicate symptoms.^(5)^ This fact, added to the determination of significant CAD by angiographic stenosis criteria and the poor value of troponins after SCA, could increase patients misdiagnosed as non-ST-segment acute coronary syndromes,^(4)^ with the inherent deleterious effects related to unnecessary PCI and medications, as well as a reduction in beneficial therapies, such as implantable cardioverterdefibrillators.

3. Coronary angiography, PCI and prognosis: Neurological damage is one of the leading causes of death in SCA, critically determining vital prognosis,^(5)^ and its severity is mainly driven by the time of the return of spontaneous circulation and early neuroprotective measures.^(5)^ It is plausible that the determination of culprit lesions by coronary angiography and an eventual PCI could have benefits in ST-segment elevation acute coronary syndromes, indicating complete acute occlusion of a coronary artery, or in cardiogenic shock scenarios but not for postanoxic encephalopathy.^(3,5)^ This investigation does not provide information regarding specific causes of death. However, we consider that reporting on them could help to adequately weigh the prognosis related to coronary angiography and PCI in SCA survivors.

In conclusion, a comprehensive assessment of CAD that globally integrates information from coronary angiography and intracoronary diagnostic techniques would allow a more precise definition of the relationship between CAD, PCI and prognosis in SCA survivors.
